# Varicocelectomy May Improve Results for Sperm Retrieval and Pregnancy Rate in Non-Obstructive Azoospermic Men

**DOI:** 10.22074/ijfs.2019.5344

**Published:** 2018-10-02

**Authors:** Hesamoddin Sajadi, Jalil Hosseini, Faramarz Farrahi, Farid Dadkhah, Mahdi Sepidarkish, Marjan Sabbaghian, Poopak Eftekhari-Yazdi, Mohammad Ali Sadighi Gilani

**Affiliations:** 1 Department of Andrology, Reproductive Biomedicine Research Center, Royan Institute for Reproductive Biomedicine, ACECR, Tehran, Iran; 2 Department of Epidemiology and Reproductive Health, Reproductive Epidemiology Research Center, Royan Institute for Reproduc- tive Biomedicine, ACECR, Tehran, Iran; 3 Department of Embryology, Reproductive Biomedicine Research Center, Royan Institute for Reproductive Biomedicine, ACECR, Tehran, Iran; 4 Department of Urology, Shariati Hosital, Tehran University of Medical Sciences, Tehran, Iran

**Keywords:** Azoospermia, Testicular Sperm Retrieval, Varicocele

## Abstract

**Background:**

Assessing the net-results of microsurgical varicocelectomy in infertile men with non-obstructive azoosper- mic (NOA) and clinical varicocele in five years at Royan Institute.

**Materials and Methods:**

This is a descriptive retrospective cohort study. A backward-looking review of patients treated for NOA and varicocele from march 2011 to march 2016 was performed. In addition, MDTESE results of 57 patients with NOA and clinical varicocele, with 537 NOA patients without varicocele were compared.

**Results:**

Of 57 patients who underwent varicocelectomy, eight patients (14%) had sperm on sperm analysis post-opera- tively. One of the eight patients was single, and one of them had spontaneous pregnancy (1/7) 14%, and one had a child by microinjection (1/7) 14%. Out of these 8 patients, 6 had hypospermatogenesis pathology. Of 38 patients who under- went MDTESE, 14 patients (36%) had sperm on their testis tissues, but one of them had no egg fertilization. Therefore, the fertilization rate was (92%). Of the remaining 13 patients, 3 had live child birth (3/13) 23%. Sperm retrieval rate (SRR) in NOA men without clinical varicocele was lower from those who had varicocele and NOA (22 vs. 36%). Also live birth rate in NOA men with varicocelectomy was higher than NOA men without varicocele (23 vs. 11%).

**Conclusion:**

Microsurgical varicocelectomy in NOA men may have positive effects on post-operative sperm in ejacu- late and natural or assisted pregnancies, but it seems that the effect is more significant on MDTESE results and follow- ing successful microinjection. Meanwhile, SRR and live birth rate was higher in our patients compare to NOA men without clinical varicocele.

## Introduction

Once infertile men with non-obstructive azoospermic (NOA) had no other options than adopting a child or using sperms of a donor to father a child, however, nowadays they are provided with other alternatives, which are given to them by the introduction of sperm retrieval from their testis and then entering an Intracytoplasmic sperm injection (ICSI) cycle (1).

Varicocele, that is associated with a progressive decline in testicular function, occurs in about 15% of total male population, 35% of men with primary infertility and between 75 to 81% of men with secondary infertility (2).

As a treatment, varicocelectomy improves both spermatogenesis and the function of Leydig cells (2). Moreover, it has been broadly reported that the density and the motility of sperms has improved in OAT patients after varicocelectomy. Nevertheless, the value of varicocelectomy is still arguable in men with azoospermia (1).

In previous studies the effects of varicocelectomy in these patients were shown to be less significant (1) but in recent studies better results have been reported for varicocelectomy in NOA patients (3-5). Therefore, several questions occur regarding sperm in the ejaculate of NOA males who will undergo varicocelectomy procedure. These questions include: are these couples able to have natural or assisted pregnancies while avoiding the need for TESE? Does varicocelectomy in these groups of patients improve SRR with MDTESE? How are the MDTESE results of these groups of patients with NOA males without varicocelectomy compared?

The present study aimed to assess the net-result of microsurgical varicocelectomy in infertile men with NOA with clinical varicocele in the past five years at Royan institute.

## Materials and Methods

This is a retrospective cohort study. A backward-looking review of patients treated for NOA and palpable varicocele in Royan institute from March 2011 to March 2016 was performed.

57 men with NOA and clinical varicocele in their physical examination have been reviewed. Known cases of obstructive azoospermia, non-palpable varicocele, female factor infertility and genetic abnormalities like klinefelter syndrome and Y-chromosome microdeletion were excluded from the study.

The cases of varicocele were identified by scrotal examinations performed by expert surgeons with the patients in standing position and during valsalva’s manoeuvre. The disease was categorized in 3 grades: grade 1 if it was palpable just during the maneuver, grade 2 if it was palpable without the maneuver and grade 3 if it was visible.

All patient charts were also reviewed for age, infertility duration, postoperation complications, testis volume, follicle-stimulating hormone (FSH), luteinizing hormone (LH), testosterone (T), testicular sonographic findings, genetic abnormalities, testicular biopsy results, sperm in ejaculate, MDTESE, fertilization rate, pregnancy and delivery rate.

In order to stay away from retrieval of testicular sperm, all NOA patients who underwent microsurgical varicocelectomy in Royan institute were inspected to find out if these patients had enough sperm in ejaculate postoperatively. Also, both assisted and unassisted pregnancy rates were evaluated using postoperative ejaculated sperm.

In addition, we have evaluated the MDTESE results in these patients and reviewed their fertilization, pregnancy and delivery rates.

Finally, we have compared the results of our 57 patients with NOA and clinical varicocele to 537 NOA patients without varicocele. All paitents in our study had been treated in Royan institute.

### Statistical analysis

For categorical and continuous variables data was reported in forms of proportions and mean ± SD, respectively. Pearson chi-square tests and Student’s t test were used to assess differences between baseline demographic and clinical characteristics. Since the sample size was small and data had several unbalanced and highly predictive risk factors (complete separation or quasi separation problems), multiple logistic regression model was performed using firthlogit to examine possible association between the outcome of interest (sperm retrieval) and microdissection TESE. The presence of the problem mentioned above in logistic regression models can result in bias in odds ratio (OR) estimates away from 1. Firthlogit command did not use maximum log likelihood but penalized log likelihood instead to reduce bias. All data analysis was completed using stata version 14 (STATACorp, College Station, TX).

## Results

For the 57 patients who were enrolled in our study from March 2011 to March 2016, the mean duration of infertility was 4.29 ± (3.97) years (range 1-12) and the mean testicular volume values were 17.36 ± (6.39) cc (range 3.2-31).

40 patients treated for varicocelectomy had karyotype analysis and azoospermia factor (AZF) microdeletion in their charts and their karyotypes were NL 46XY and no one had microdeletion.

Of the 57 patients, 8 (14.03%) acquire motile sperms in a postoperative sperm analysis. Of these 8 patients, 6 had hypospermatogenesis, 1 had maturation arrest and 1 had sertoli only syndrome (SOS) in histopathology.

One of the patients was single, and one of them had spontaneous pregnancy (1/7) 14%, and one had children through microinjection (1/7) 14%.

Microdissection TESE was applied to 38 (66.7) NOA patients, who had negative sperm postoperatively (Table 1). The mean interval between varicocelectomy and microTESE was 13.7 months (range 3 to 17). Prior to operation varicocele grades were 1, 2, and 3 in 8 (21.05%), 16 (42.1%) and 14 (36.84%) of these patients, respectively. Of 38 patients who underwent MDTESE, 14 (36.8%) had sperm in their testis tissues. Of these 14 patients, 8 had maturation arrest, 3 had hypospermatogenesis and 3 had SOS in their biopsies. In addition, of these 14 patients one had no egg fertilization, therefore, the fertilization rate was 13 (92%).

A total of 530 patients with NOA without varicocele were selected as the control group. Characteristics of the control group are described in Table 1. The mean ± SD patient age was 33.84 ± (7.27) years for the cases and 34.10 ± (6.35) years for the controls (P=0.810). The controls had a significantly higher FSH [22.48 ± (14.47)] (mIU/mL) compared to the case group [17.50 ± (16.65)] (P=0.05). Base on other laboratory parameters, no significant difference was observed between these two groups.

Sperm retrieval rate by MDTESE in the cases and the control groups were 14/38 (36.8%) and 119/530 (22.3%), respectively (OR=2.03, 95% CI: 1.01-4.05, P=0.041). The live birth rate in the cases and the control groups was 21.42% (3 of 14 cases) and 11.7% (14 of 119 controls), respectively (OR=2.21, 95% CI: 0.59-8.14, P=0.219).

**Table 1:** Characteristics of patients


Parameter	Cases	Controls	P value

Age (Y)	33.84 ± 7.27	34.10 ± 6.35	0.810
FSH (mIU/mL)	17.50 ± 16.65	22.48 ± 14.47	0.05
LH (mIU/mL)	8.44 ± 6.80	8.84 ± 7.18	0.743
Testosterone (ng/ml)	3.20 ± 2.38	3.48 ± 2.23	0.477
SRR	14(36.8)	119(22.3)	0.410
Live birth rate	3(21.42)	14(11.7)	0.219


Values given as mean ± SD, or n(%) unless otherwise indicated. FSH; Follicular stimulating hormone, LH; Luteinizing hormone, and SRR; Sperm retrieval rate.

## Discussion

While the impact of varicocelectomy has been widely considered in oligoasthenoteratozoospermia (OAT) patients, this surgical procedure’s benefit in patients with NOA is limited and still arguable.

Tulloch was the first who studied the importance of varicocelectomy for treatment of NOA in 1952 (6). After that, many studies investigated the effect of this surgical procedure on NOA patients.

From 57 patients who underwent varicocelectomy at Royan institute, eight patients (14%) had sperm on sperm analysis postoperatively and only one of the patients had spontaneous pregnancy. So in terms of postoperative sperm in ejaculate the effect of varicocelectomy in our patients was little, and this result was nearly the same as the findings of the study by Shlegele et al. (22%) (1). Other studies have reported variable results from 34 to 44% (3-5).

So far, studies report testicular histology as one of the most important predictor factor outcomes (7-9). Our data support this as the most histopathological predictor of postoperative sperm in the ejaculate was hypospermatogenesis: from 8 patients who had achieved motile sperm postoperatively, 6 had hypospermatogenesis.

Sperm retrival rate by MDTES was (36/8%) in NOA men with varicocelectomy compared to (22%) in NOA cases without varicocele. Therefore, varicocelectomy in NOA men with varicocele may improve the chance of SRR compared to NOA men without varicocele.

Live birth rate/embryo was 21% in our patients in comparison with 11% in NOA without varicocele.

## Conclusion

According to our current data, we suggest that microsurgical varicocelectomy in NOA patients may have positive effects on postoperative sperm in the ejaculate and spontaneous or assisted pregnancies, but it seems that this effect is more significant on MDTESE results when following successful microinjection. Meanwhile, SRR and live birth rate were higher in our patients compared to NOA males without clinical varicocele. The most histopathologic finding in microTESE-positive patients was maturation arrest, which shows the need of MDTESE for sperm retrieval in such patients.

Thus, varicocelectomy is a key factor in NOA patients to increase the likehood of SRR in MDTESE. Nevertheless, studies with a larger population and a longer follow-up period are needed in order to prove MDTESE benefits in these patients.

## References

[1] Schlegel PN, Kaufman J (2004). Role of varicocelectomy in men with nonobstructive azoospermia. Fertil Steril.

[2] Wein AJ, kavoussi LR, Partin AW, Peters CA (2015). Campbell Walsh urology.11th edition.

[3] Youssef T, Abd-Elaal E, Gaballah G, Elhanbly S, Eldosoky E (2009). Varicocelectomy in men with nonobstructive azoospermia: is it beneficial?. Int J Surg.

[4] Zampieri N, Bosaro L, Costantini C, Zaffagnini S, Zampieri G (2013). Relationship between testicular sperm extraction and varicocelectomy in patients with varicocele and nonobstructive azoospermia. Urology.

[5] Esteves SC, Miyaoka R, Roque M, Agarwal A (2016). Outcome of varicocele repair in men with nonobstructive azoospermia: systematic review and meta-analysis. Asian J Androl.

[6] Tulloch WS (1951-1952). A consideration of sterility factors in the light of subsequent pregnancies.II.Sub fertility in the male.(Tr.Edinburgh Obst.Soc.Session 104). Edinb Med J.

[7] Matthews GJ, Matthews ED, Goldstein M (1998). Induction of spermatogenesis and achievement of pregnancy after microsurgical varicocelectomy in men with azoospermia and severe oligoasthenospermia. Fertil Steril.

[8] Kim ED, Leibman BB, Grinbat DM, Lipshultz LI (1999). Varicocele repair improve semen parameters in azoosperic men with spermatogenic failure. J Urol.

[9] Kadioglu A, Tefkeli A, Cayan S, Kendirali E, Erdemie F, Tellaloglu S (2001). Microvascular inguinal varicocele repair in azoospermic men. Urology.

